# Modeling production curves in a strawberry breeding program to optimize early season productivity

**DOI:** 10.3389/fpls.2026.1808529

**Published:** 2026-07-16

**Authors:** Sehijpreet Kaur, Joshua Sleper, Cheryl Dalid, Julian Garcia-Abadillo, Vance M. Whitaker, Diego Jarquin

**Affiliations:** 1 Agronomy Department, University of Florida, Gainesville, FL, United States; 2 Gulf Coast Research and Education Center, University of Florida, Gainesville, FL, United States

**Keywords:** forward prediction, genomic selection, longitudinal modeling, marketable yield, strawberries (*Fragaria x ananassa* Duch.), Delta Yield

## Abstract

Florida and California produce 98% of U.S. strawberries, with Florida growers’ profitability depending on high yields early in the season (November-January), when prices are the highest in the U.S. market. This study aims to model the cumulative Marketable Yield and Delta Yield curves (difference in cumulative Marketable Yield between consecutive harvest time points) of strawberry genotypes to facilitate selection for greater early season productivity. The dataset comprised thirteen seasons (2013–14 to 2025-26) of advanced selection trial data. Marketable Yield trajectories were modeled using Legendre polynomial smoothing, with optimal degree selection balancing flexibility and noise reduction. The resulting coefficients served as surrogate phenotypes for genomic prediction. Forward prediction cross-validation was implemented for five seasons (2021-22, 2022-23, 2023-24, 2024-25, and 2025-26), with each season predicted using the information from all preceding seasons. For cumulative yield, across all five seasons, reconstructed curves from the Legendre models presented a clear temporal trend, with predictive ability increasing from low early-season values to peaks around Trait Dates (weeks) 7-8. In the 2021–22 and 2022–23 seasons, Legendre models showed higher predictive ability than single time-point predictions but were comparable to single-time point predictions for the other seasons. Legendre polynomial models utilizing Delta Yield achieved moderate predictive ability across five validation seasons, with consistent advantages over single time point models particularly in earlier seasons, indicating that genetic control extends beyond total yield to the trajectory of yield accumulation. Overall, Legendre modeling effectively captured the temporal dynamics of yield development while describing the trajectory with only a few parameters.

## Introduction

Fresh strawberries (*Fragaria × ananassa* Duch.) are an important fruit crop in the U.S., with central Florida being the primary source of winter strawberry production in the eastern U.S. due to its sub-tropical climate. Rising input costs and competition from imports have reduced profit margins in the U.S. strawberry industry, making early-season yield gains increasingly important for growers in Florida (Guan et al., 2018). Because strawberries are highly perishable, supply can be volatile causing direct and strong effect on market prices. North American supply of fresh strawberries is lowest during November and December resulting in a market premium for Florida strawberries. Wu et al. (2015) derived an optimal strawberry yield distribution by price-response analysis that maximizes the profit to Florida growers. Their results showed that yield increases during November and December have the largest effect on gross margin while maintaining stable productivity during the rest of season helps prevent market oversupply.

For these economic reasons, breeding strategies for the Florida strawberry industry should focus on an optimum yield distribution as opposed to total yield throughout the season, prioritizing genetic gains for early season yield. Achieving these objectives requires breeding tools that can accurately predict performance at specific points within the production season. To address this need, genomic prediction has emerged as a powerful tool (Bernardo, 1994; Meuwissen et al., 2001). It leverages the relationship between phenotype and genotype using genetic marker and phenotypic information. The models can then be used to predict the phenotypes of unobserved individuals. Implementing genomic prediction early in breeding cycle allows for accelerated selection, increased selection intensity, and more efficient resource allocation, ultimately leading to greater genetic gains.

Different strawberry genotypes exhibit distinct yield patterns throughout the growing season. The plant’s response to environmental changes is dynamic and has a strong temporal component, with the mean and covariance of yield observations across time points continuously changing (Momen et al., 2019). Measurements taken close in time are more correlated because plant growth and yield accumulation are gradual, while wider time intervals capture greater physiological and environmental changes, reducing correlation. Early in the season fruiting is low for all genotypes, but as yield accumulates, genetic and environmental differences amplify, increasing both the mean and variance of observations. This temporal variation is further shaped by the characteristic multi-wave fruiting pattern of strawberry, in which weekly yields fluctuate substantially in response to temperature and plant development cycles (MacKenzie and Chandler, 2009; Pinochet and Agehara, 2024). That genotypes differ in their yield trajectory across the season is not merely a phenotypic observation; Shaw (1989) demonstrated that broad- and narrow-sense heritabilities for strawberry yield follow distinct seasonal patterns, indicating temporal expression of genes conditioning yield accumulation. Single time point measurements capture only a snapshot of yield, overlooking temporal variation that is critical for identifying promising genotypes. Delta Yield, defined as the difference between consecutive cumulative yield measurements, reflects the rate of yield accumulation and the week-to-week volatility that distinguishes genotypes across the harvest time-series. In crops where fruiting occurs multiple times, repeated measurements are essential to accurately characterize yield potential, and integrating this temporal element into genomic prediction has the potential to increase genetic gain.

Genomic selection (GS) using longitudinal data has been explored through various methodologies. Repeatability models treat repeated measurements as the same trait but assume equal variance and constant correlation across time, assumptions rarely met for complex longitudinal traits (Sun et al., 2017). Multivariate approaches extend GS to account for time-dependent variation but become computationally prohibitive as the number of time points increases (Klápště et al., 2020). Random regression models address these limitations by fitting basis functions to longitudinal trajectories, allowing genetic (co)variances to change over time while reducing dimensionality (Campbell et al., 2018). Factor-analytic methods, such as MegaLMM (Runcie et al., 2021), further reduce dimensionality by capturing temporal structure through latent factors, scaling well to high-frequency data. Despite these advances, a key challenge remains in representing longitudinal yield trajectories in a low dimensional form that is compatible with standard genomic prediction pipelines. Existing approaches such as random regression models require estimation of time-dependent (co)variance structures, while factor-analytic methods like MegaLMM rely on latent factors that can be less directly interpretable. These limitations highlight the need for approaches that balance dimensionality reduction, interpretability, and computational efficiency.

To better capture temporal dependencies, researchers also apply smoothing or parametric functions. Orthogonal polynomials, especially Legendre polynomials, are widely used for biological curves (Albuquerque and Meyer, 2001; de Oliveira et al., 2017). These functions efficiently represent trait variation over time, offer flexible order selection for curve fitting, and remain linear in parameters despite being nonlinear in time (Yang et al., 2006).

The aim of this study is to assess the effectiveness of genomic prediction models utilizing Legendre polynomial coefficients derived from longitudinal Marketable Yield data, including both cumulative yield and the incremental change in cumulative yield (hereafter referred to as *Delta Yield*), in comparison to the single time point predictions. Specifically, this study aims to *i*) develop and evaluate genomic prediction models using Legendre polynomial coefficients derived from longitudinal yield data, *ii*) estimate time-specific heritability and assess prediction ability across the growing season, and *iii*) compare the performance of genomic prediction using longitudinal Legendre-based models to single time-points. By summarizing temporal yield trajectories into a small set of coefficients, this approach enables dimensionality reduction and facilitates the integration of longitudinal information into standard genomic prediction frameworks while maintaining interpretability of temporal patterns.

## Materials and methodology

### Phenotypic data

**Figure 1:**
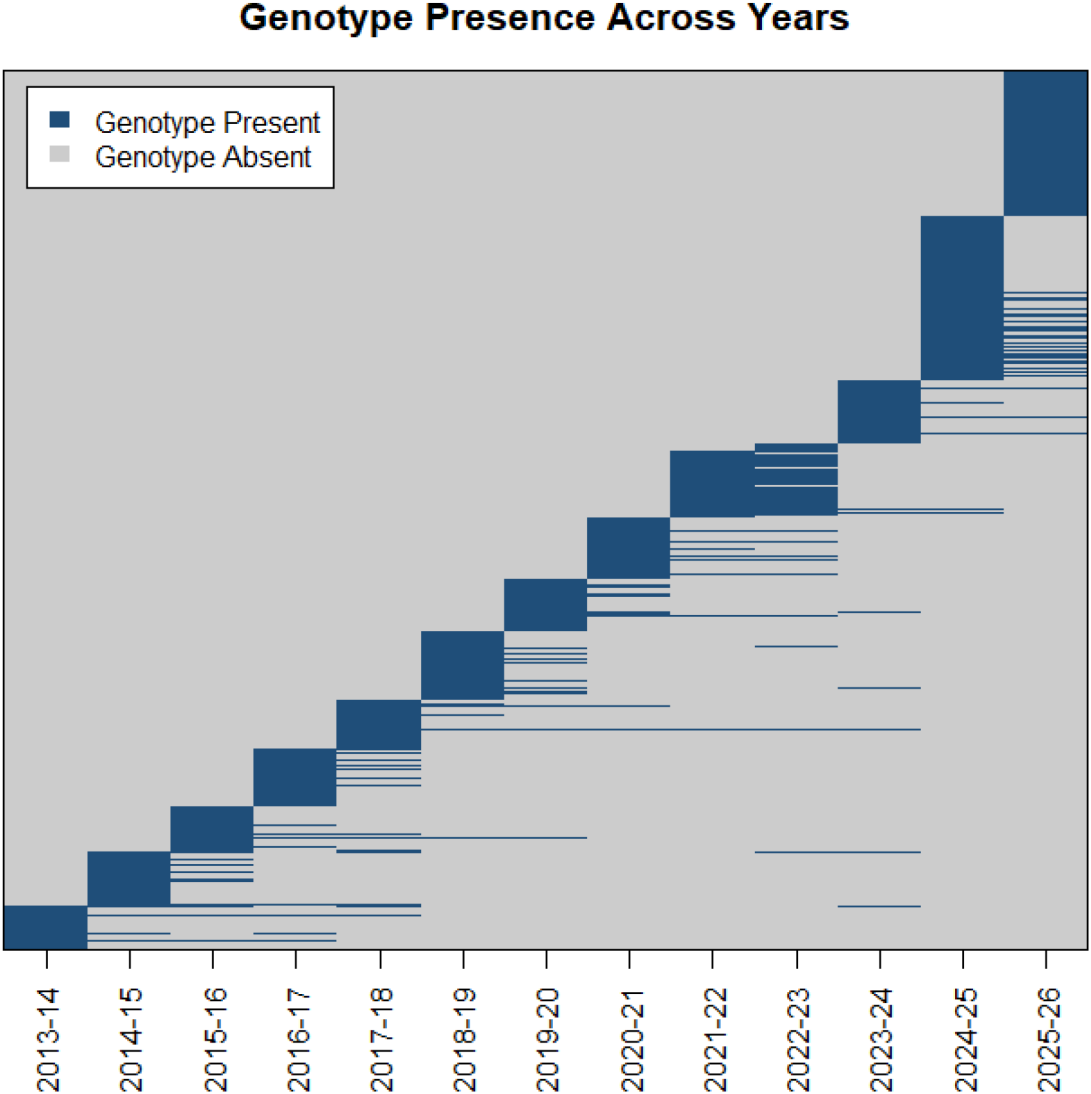
Distribution of genotypes across different years. The *x*-axis presents the different evaluation seasons, and the *y*-axis represents the unique genotypes. Blue/gray colored horizontal lines correspond to observed/unobserved genotypes in the corresponding seasons.

This study utilized a large strawberry breeding dataset collected over thirteen growing seasons from 2013–2014 to 2025–2026 at the UF/IFAS Gulf Coast Research and Education Center (GCREC), Balm, Florida, USA (27° 45′ 37.98″ N, 82° 13′ 32.49″ W). The University of Florida strawberry breeding program primarily operates as a single recurrent selection population established through partial half-diallel mating designs (Osorio et al., 2021), with relatively short breeding cycles of 3–4 years that minimize population structure (Alam et al., 2024). Across the thirteen years of evaluation, a total of 4,492 unique strawberry genotypes were evaluated. Each breeding cycle included both genotypes retained from previous seasons and newly introduced lines. On average, approximately 15% of genotypes overlapped between consecutive years (**Figure 1**). The number of genotypes per cycle ranged from 226 to 1013. The experiments were grown in a randomized complete block design (RCBD) with five replications except in the 2024–25 and 2025–26 seasons, which included two and three replications, respectively. Each replication consisted of two beds and each bed was subdivided into five to eight plots, with a designated cultivar check included in each plot. Plots were arranged using a row-column layout to account for field heterogeneity. Each replication consisted of a single plant representing one genotype. Because strawberry plants are clonally propagated, the plant in each replication represented a clonal replicate of the same genotype. The weight of the marketable fruit in grams (“Marketable Yield”) was recorded weekly for each genotype in each replicate throughout the growing season. Within each season, cumulative Marketable Yield was calculated by adding weekly harvest weights, providing an integrated measure of productivity. In addition, Delta Yield was derived as the week-to-week difference in cumulative marketable yield, capturing incremental changes in productivity and offering a dynamic representation of yield accumulation across the growing season.

### Statistical model for BLUEs

To minimize spatial variability and obtain a representative estimate of genotype performance, best linear unbiased estimates (BLUEs) were computed separately for each genotype at each time point within each season by accounting for experimental design factors, including replicate, row, and column effects. The following linear mixed model was fitted


$$y_{ijkl}=\mu +L_{i}+Rep_{j}+Row_{k}+Col_{l}+\epsilon_{ijkl}$$

where $y_{ijkl}\;$is the observed Marketable Yield of the $i^{th}$ ( $L_{i}$) genotype measured in $j^{th}$ replicate ( $Rep_{j}$), $k^{th}$ row ( $Row_{k}$), and $l^{th}$ column ( $Col_{l}$) at a given time point within a harvest season. The overall mean is represented by μ, $L_{i}$ is the fixed effect of the $i^{th}$ genotype, $Rep_{j}$ represents the random effect of the $j^{th}$ replication assumed to follow $Rep_{j}∼N(0,\;\sigma_{rep}^{2})$, $Row_{k}\;$is the random effect of $k^{th}$ row modeled as $Row_{k}∼N(0,\sigma_{row}^{2})$, $Col_{l}\;$is the random effect of $l^{th}$ column assumed as $Col_{l}∼N(0,\;\sigma_{col}^{2})$, $\epsilon_{ijkl}$ represents the residual error such that $\epsilon_{ijkl}∼N(0,\;\sigma^{2})$; where $\sigma_{rep}^{2}$, $\sigma_{row}^{2}$ $\sigma_{col}^{2}$, and $\sigma^{2}$ are the corresponding variance components.

This approach leveraged the structure of the field trial to generate adjusted yield values that more accurately reflect the true performance of each genotype. For reliable curve reconstruction, a subset of genotypes consistently planted across seasons and with sufficient within-season records was retained for analysis. Following the estimation of BLUEs, Delta Yield was computed as the week-to-week difference in cumulative marketable yield to capture incremental changes in productivity across the growing season. To facilitate comparative analysis across seasons and time points, data were aligned based on season-specific time scale (days after planting), ensuring consistency in evaluating temporal yield trends.

### Selection of random regression model

Legendre polynomials are a set of orthogonal polynomials widely used in random regression models to model longitudinal variation (Meyer, 1998; Kirkpatrick et al., 1990). These polynomials provide a flexible approach for capturing phenotypic changes over time. The Legendre polynomial of degree *n*, denoted as $P_{n}(x)$, can be defined using Rodrigues’ formula (Altmann and Ortiz, 2005) as


$$P_{n}(x)=\frac{1}{2^{\text{n}}n!}\frac{d^{\text{n}}}{dx^{\text{n}}}\;{\left(x^{2}-1\right)}^{\text{n}}$$

Within each season, a Legendre polynomial was fit for each genotype up to the eighth week of the harvest season, which was approximately ninety-five days after planting, covering the period through mid-January (**Supplementary Table 1**). The eight time points encompassed early establishment, mid establishment, and early maturity stages, ranging from 49 to 98 days after planting. The within-season polynomial allowed for year-specific environmental effects which was necessary due to variation in planting and first harvest dates across seasons. Planting dates in the dataset ranged from late September to mid-October, reflecting seasonal and operational variation across years. Correspondingly, the first harvest dates also varied by season, highlighting the influence of planting time on crop development. To determine the optimal polynomial degree, the Mean Squared Error (MSE) and the correlation between the predicted and the observed values were used as the primary selection criterion. Within each season, for all genotypes, the observed time periods corresponding to approximately 90–95 days after planting and their corresponding estimated values obtained using the Legendre polynomial were stacked across all genotypes, and a single Pearson correlation value between these two vectors was used as the yearly accuracy estimate. A third-degree Legendre polynomial was chosen, as it provided a lower MSE and higher correlation across datasets while maintaining computational efficiency. Hence, within a season for each individual, phenotypic values $\left(y_{i}\right)$ across time points were modeled using the third-degree Legendre polynomial as


$$y_{i}=\beta_{0}P_{0}(x)+\;\beta_{1}P_{1}(x)+\;\beta_{2}P_{2}(x)+\;\beta_{3}P_{3}(x)+e_{i}\;$$

where $y_{i}$ represents the phenotype of the $i^{th}\;$individual; $\beta_{0},\;\beta_{1},\;\beta_{2},\;$ and $\;\beta_{3}$ are the estimated regression coefficients and $\;P_{n}(x)$ are the basis functions derived from Rodrigues` formula with $P_{0}(x)=1,\;P_{1}(x)=x,\;P_{2}(x)=\frac{1}{2}(3x^{2}-1),\;P_{3}(x)=\frac{1}{2}(5x^{3}-3x)$ and $e_{i}$ is the residual error term such that $e∼N(0,\;\sigma_{e}^{2})$.

After fitting the Legendre polynomial model, the regression coefficients were extracted. These coefficients represent the underlying growth dynamics of the trait over time, effectively reducing the dimensionality of the dataset while preserving key information about temporal variation. Legendre basis functions capture the temporal structure of the trait, allowing the resulting coefficients to serve as compact descriptors of yield trajectories; these coefficients were modeled independently to enable a practical and computationally efficient genomic prediction framework, without explicitly modeling covariance among coefficients.

### Genotypic data and quality control

Genotyping was carried out using either the iStraw35 K SNP array (Bassil et al., 2015) or the Fana50 K SNP array (Hardigan et al., 2020). Imputation was done with PolyBreedR (Sleper et al., 2017). Genotypes were recoded to an additive scale, where each marker was coded as 0, 1, or 2, representing the number of alternate alleles. Two marker-level quality control filters were applied. SNPs with more than 20% missing data and less than a 5% minor allele frequency (MAF) were removed. After applying these filters, the resulting marker matrix consisted of 42,543 SNPs and these were exported for downstream analyses.

### Genomic prediction models

Within each season, the different coefficients resulting from the Legendre polynomial were treated as a separate traits (s=1,2,3,4) thus were modeled independently using the following two genomic prediction models. These models were implemented for both cumulative marketable yield and Delta Yield.

### M1: genotypic, marker, and environmental main effect

Model M1 includes only the main effects of genotypes and environments (season). In this specification, phenotypic variation in each coefficient is explained by additive genetic effects and by overall environmental differences. This model is represented as


$$c_{ijs}=\mu +l_{is}+{\text{g}}_{is}+\;E_{js}+\epsilon_{ijs}$$

where $c_{ijs}$ represents the estimated $s^{th}$ coefficient for the $i^{th}\;$genotype in the $j^{th}$ environment , μ is the corresponding overall mean, $l_{is}\;$represents the line effect and was modeled as a random effect following $l_{is}∼N(0,\sigma_{l}^{2}),\;$ ${\text{g}}_{is}\;$represents the genomic effect of $i^{th}$ genotype characterized by the main effect of the markers (SNPs) such that ${\text{g}}_{is}=\sum_{m=1}^{p}x_{ism}b_{sm}$ with $b_{sm}∼N(0,\sigma_{b_{s}}^{2})$ where $\sigma_{b_{s}}^{2}$ is the corresponding variance component. Collecting the previous results, we have that the vector of genomic effects $\;g=\{{\text{g}}_{is}\}∼N(0,\;G\sigma_{g_{s}}^{2})\;$where $G=\frac{XX^{\prime }}{p}$ represents the genomic relationship matrix (GRM) whose cells describe the genomic similarities between pairs of genotypes, X is the centered and standardized matrix of molecular markers and p is the number of markers and $\sigma_{g_{s}}^{2}=p\times \sigma_{b_{s}}^{2}$ is the genetic variance of the genotypes for $s^{th}$ coefficient; $E_{js}$ represents the random effect of $j^{th}$ environment for $t^{th}$ coefficient and it is also assumed to follow independent and identically distributed multivariate normal distribution such that $E_{js}∼N(0,\;\sigma_{E_{s}}^{2})$; $\;\epsilon_{ijs}$ represents the random error with $\epsilon_{ijs}∼\;N(0,\sigma_{e}^{2})$ where $\sigma_{e}^{2}$ is the residual variance.

### M2: genotypic, marker, environmental, and marker by environment [G × E] interaction model

To model genotype-specific responses to different environmental conditions, M1 can be extended by incorporating the genotype by environment interaction term (Jarquín et al., 2014). This model is represented as


$$c_{ijs}=\mu +1_{is}+\;{\text{g}}_{is}+E_{js}+\text{g}E_{ijs}+\epsilon_{ijs}$$

where $\text{g}E_{ijs}$ represents the interaction effect between the *i*^th^ genotype and the *j*^th^ environment which is assumed to follow multivariate normal distribution such that  gE={gEijt}∼N(0,[Zg GZg']°[ZEZE']σgEt2), where $Z_{\text{g}}$ and $Z_{E}$ are the incidence matrices that respectively connect phenotypic responses to genotypes and environments, $\sigma_{\text{g}E_{s}}^{2}$ is the associated variance component and ° denotes the Hadamar or Schur product (element-by-element product) between two matrices.

### Cross validation scheme

To assess the predictive performance of genomic prediction models, a forward-prediction cross-validation scheme was implemented. In this approach, model training relied only on historical seasons, and predictions were generated for the next season in chronological order. The size of training dataset increased over time as additional seasons became available.

**Figure 2:**
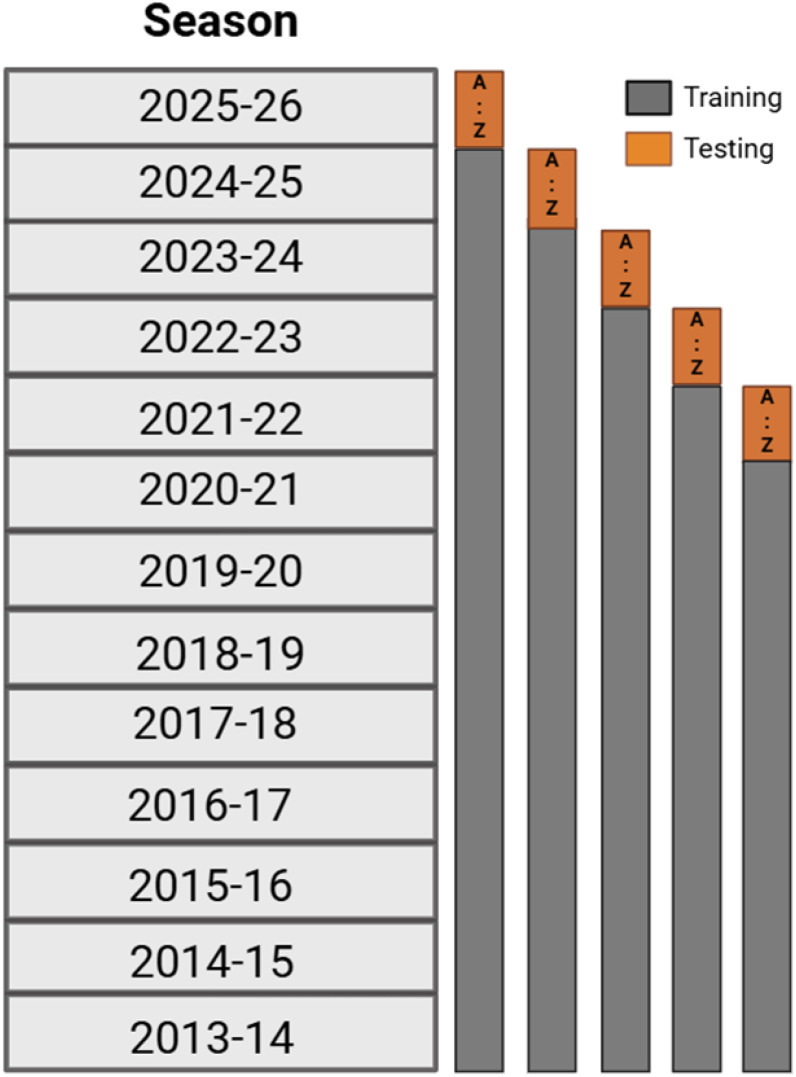
Cross-validation scenario used for evaluating genomic prediction models under forward-prediction settings.

Five recent seasons (2021-22, 2022-23, 2023-24, 2024-25, and 2025-26) were treated as independent validation years. For each validation year, predictions were made using all preceding seasons as the training dataset. Specifically, predictions for the 2021–22 season were based on data from the eight preceding seasons (2013–14 to 2020-21). Predictions for 2022-23, 2023-24, 2024-25, and 2025–26 were generated using data from the nine, ten, eleven, and twelve preceding seasons, respectively (**Figure 2**).

### Transformation of predicted Legendre coefficients

After implementing genomic prediction models, the predicted Legendre polynomial coefficients were used to reconstruct the original production curves and to estimate the predicted cumulative yield values over time during the harvest season. The reconstruction was performed using the polynomial expansion


$$\hat{y}(x)=\sum_{t=1}^{T}{\hat{\beta }}_{t}P_{t}(x)$$

where $\hat{y}(x)$ is the estimated trait value at the x time point, ${\hat{\beta }}_{t}$ are the predicted Legendre coefficients, and $P_{t}(x)$ are the corresponding Legendre polynomial basis functions of order t evaluated at x. The trait curve at each time point is reconstructed by combining these weighted polynomial functions, with the coefficients ${\hat{\beta }}_{t}$ determining the contribution of each polynomial term to the overall curve. This reconstruction models the trait response along the harvest season using few parameters in function of the time. The reconstructed values will represent the target trait response at the timepoints of interest (harvests dates) (**Supplementary Figure 1**).

### Single time point genomic prediction approach

To evaluate the benefit of using Legendre polynomials for modeling yield trajectories, the results of this framework were compared to a more traditional genomic prediction approach that treats cumulative Marketable Yield and Delta Yield at different time points as independent traits.

In the traditional approach, cumulative Marketable Yield and Delta Yield at each Trait Date (weeks 1–8) was analyzed separately. For each Trait Date, cumulative Marketable Yield was considered an independent trait, and the genomic prediction models (M1 and M2) were fit for each time point. Predictive ability was estimated for each Trait Date under the single time point prediction framework across the five validation seasons. In contrast, the Legendre polynomial framework modeled both cumulative Marketable Yield and Delta Yield as a continuous function of time across the season. This approach jointly estimated yield trajectories by capturing temporal covariance among repeated harvest measurements, allowing information from multiple time points to contribute to the prediction of yield throughout the season.

Predictive performance from both approaches was evaluated across five validation seasons using identical training populations and cross-validation schemes. Comparisons were made at corresponding Trait Dates to determine whether incorporating temporal structure through Legendre polynomials improved prediction ability relative to the conventional, week-by-week modeling strategy.

### Assessing predictive ability

Predictive ability was used to evaluate model performance. Predictive ability was evaluated at two levels: *i*) parameter level $\left(r_{bp}\right)$, as the correlation between the observed Legendre coefficients (β) and those predicted using GS $(\hat{\beta }),\;$ and *ii*) phenotype level ( $r_{yp}$), as the correlation between the observed phenotypic values ( y)and the predicted phenotypic value ( $\hat{y}).$ The observed phenotypes were obtained by reconstructing yield curves using the estimated Legendre coefficients, while predicted phenotypes were obtained by reconstructing curves using the predicted Legendre coefficients:


$$r_{bp}=cor(\beta ,\;\hat{\beta })$$

$$r_{yp}=cor(y,\hat{y})$$

For the 2021-22, 2022-23, 2023-24, 2024-25, and 2025–26 seasons, the narrow sense heritability ( $h^{2}$) was calculated for each coefficient and Marketable Yield for each one of the eight time points to quantify the genetic contribution to phenotypic variation


$$h^{2}=\frac{\sigma_{g}^{2}}{\sigma_{g}^{2}+\sigma_{e}^{2}}$$

where $\sigma_{g}^{2}$ represents additive genetic variance and $\sigma_{e}^{2}$ is the residual variance. Heritability was estimated independently for each season and each trait date to avoid confounding effects arising from the unbalanced multi-year structure of the dataset.

The standard error of heritability was derived using the asymptotic formulation based on the eigenvalues $(\lambda_{i})$ of the genomic relationship matrix (Visscher and Goddard, 2015). Specifically,


$$a=\sum_{i}\frac{{\left(\lambda_{i}-1\right)}^{2}}{{\left(1+h^{2}\left(\lambda_{i}-1\right)\right)}^{2}}$$

$$b=\sum_{i}\frac{\lambda_{i}-1}{\left(1+h^{2}\left(\lambda_{i}-1\right)\right)}$$

$$SE(h^{2})=\sqrt{\frac{2}{a-\frac{b^{2}}{n}}}$$

Coincidence index was used to quantify the concordance between the top-ranked genotypes identified from observed values and those identified from model predictions. For each timepoint, genotypes were ranked by observed yield and by predicted yield, and the overlap between the top 30% selected genotypes was calculated. The coincidence index was calculated as the proportion of genotypes shared between the observed and predicted top-selected groups relative to the total number selected for Delta Yield.

## Results

### Selection of polynomial degree

**Figure 3:**
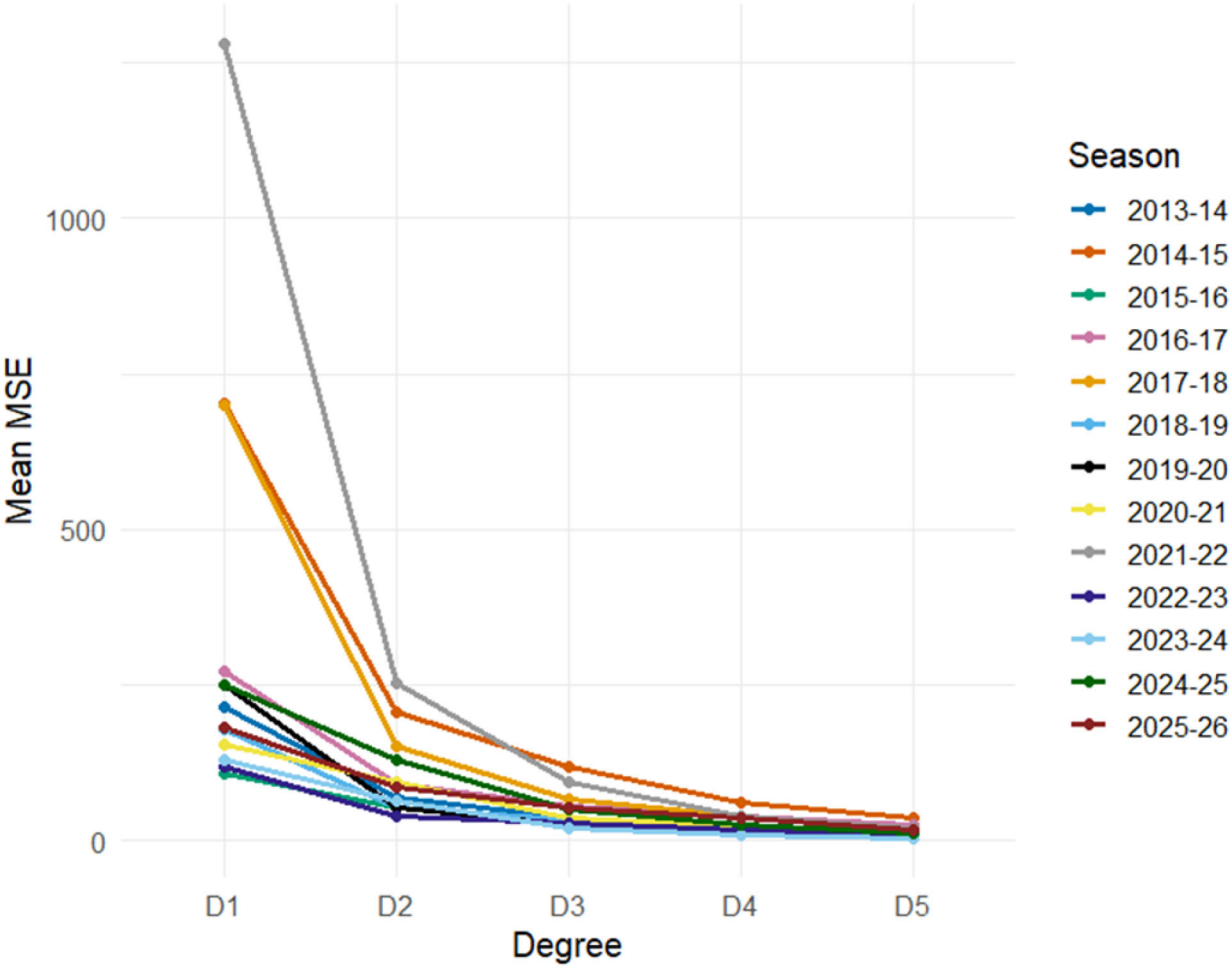
Mean squared error across polynomial degrees (D1–D5) for each season.

Across all the environments, increasing polynomial degree consistently reduced MSE while improving correlation between observed and estimated values from Legendre polynomial. The first order polynomial produced relatively poor fits, with MSE ranging 130.7-1,316 and correlations 0.88-0.99 across different years. Adding a quadratic term (degree 2) substantially improved the performance, reducing MSE by more than half in most years (43.6-268.4) and increasing correlations between observed and estimated values from Legendre polynomial model above 0.95 (**Figure 3**).

Degree 3 polynomial further improved fit, yielding a lower MSE values in nearly every season (18.7-114.6) maintaining very high correlations (0.99). While degree 4 models produced even lower MSE, it did not improve correlation with the observed data compared to degree 3 polynomial. Degree 3 was ~99% as effective as degree 4 in terms of correlation. In contrast, adding the fourth term increased the number of parameters to estimate and risk of overfitting, especially given the limited number of time points used within each season. To balance model fit and parsimony, the analysis was restricted to degree 3.

### Heritability of cumulative marketable yield and its Legendre coefficients

**Figure 4:**
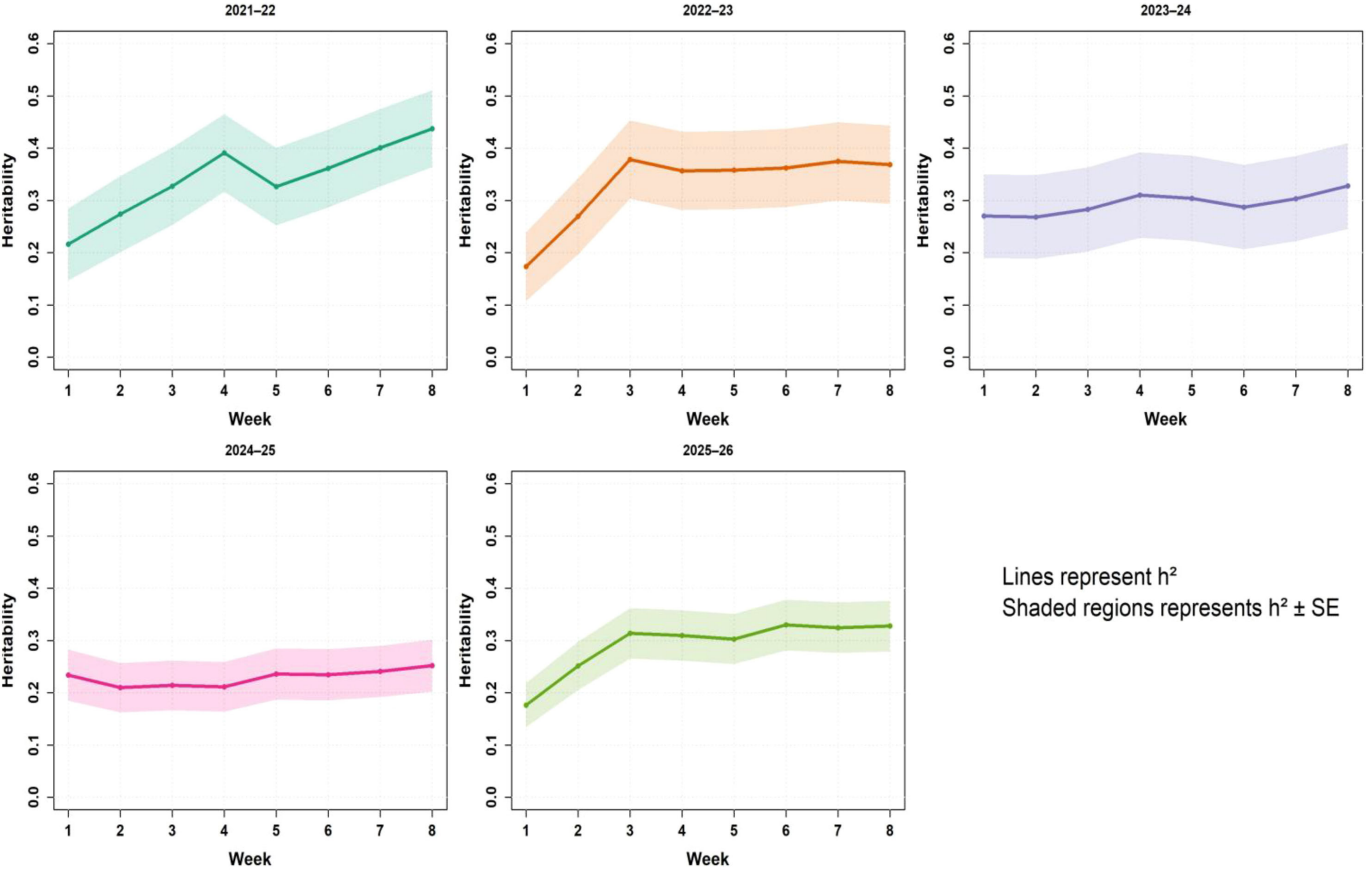
Heritability (H²) of cumulative marketable yield across trait dates (weeks 1–8 of fruiting) for the 2021–22, 2022–23, 2023–24, 2024–25, and 2025–26 season. Shaded regions represent heritability ± Standard Error ( $h^{2}\;\pm \;SE)$.

The intercept ( $\beta_{0}$) and first-order coefficient ( $\beta_{1}$) showed the highest heritability across seasons, with estimates ranging from 0.22 to 0.41 for $\beta_{0}$ and 0.26 to 0.44 for $\beta_{1}$ (**Table 1**). The higher-order coefficients $\beta_{2}$ and $\beta_{3}$ displayed lower values compared to lower order coefficients, ranging from 0.17 to 0.48 for $\beta_{2}$ and 0.20 to 0.31 for $\beta_{3}$ (**Table 1**). The heritability of cumulative Marketable Yield across Trait Dates 1 to 8 for the five seasons is shown in **Figure 4**. In 2021-22, estimates increased from approximately 0.21 at Trait Date 1 to 0.44 at Trait Date 8, with a peak around 0.39 at Trait Date 4. In 2022-23, heritability values ranged from 0.17 to 0.37, with the highest estimate observed at Trait Date 3 followed by stable values between 0.34 and 0.38 during the later Trait Dates. For 2023-24, estimates were lower overall, varying from 0.27 to 0.33, with a gradual increase across the season and slight fluctuations in mid-season. In 2024-25, heritability values remained stable across trait dates ranging from 0.21to 0.25. In 2025-26, estimates were low (0.18) at early Trait Dates and increased to 0.33 as season progressed. Across all seasons, heritability generally increased from early to later Trait Dates, with modest differences in magnitude and trajectory among years.

**Table 1:** Narrow-sense heritability (H²) of Legendre polynomial coefficients ( $\beta_{0},\;\beta_{1},\;\beta_{2},\;and\;\beta_{3}$) describing temporal cumulative yield trajectories across the 2021–22, 2022–23, 2023–24, 2024–25, and 2025–26 season.

Season	Narrow sense heritability			
	$\beta_{0}$	$\beta_{1}$	$\beta_{2}$	$\beta_{3}$
2021-22	0.41	0.44	0.48	0.31
2022-23	0.40	0.34	0.23	0.20
2023-24	0.31	0.32	0.26	0.24
2024-25	0.22	0.26	0.23	0.22
2025-26	0.35	0.29	0.17	0.22

### Predictive performance of Legendre polynomial and single-time-point models

#### (i) Cumulative marketable yield

**Figure 5:**
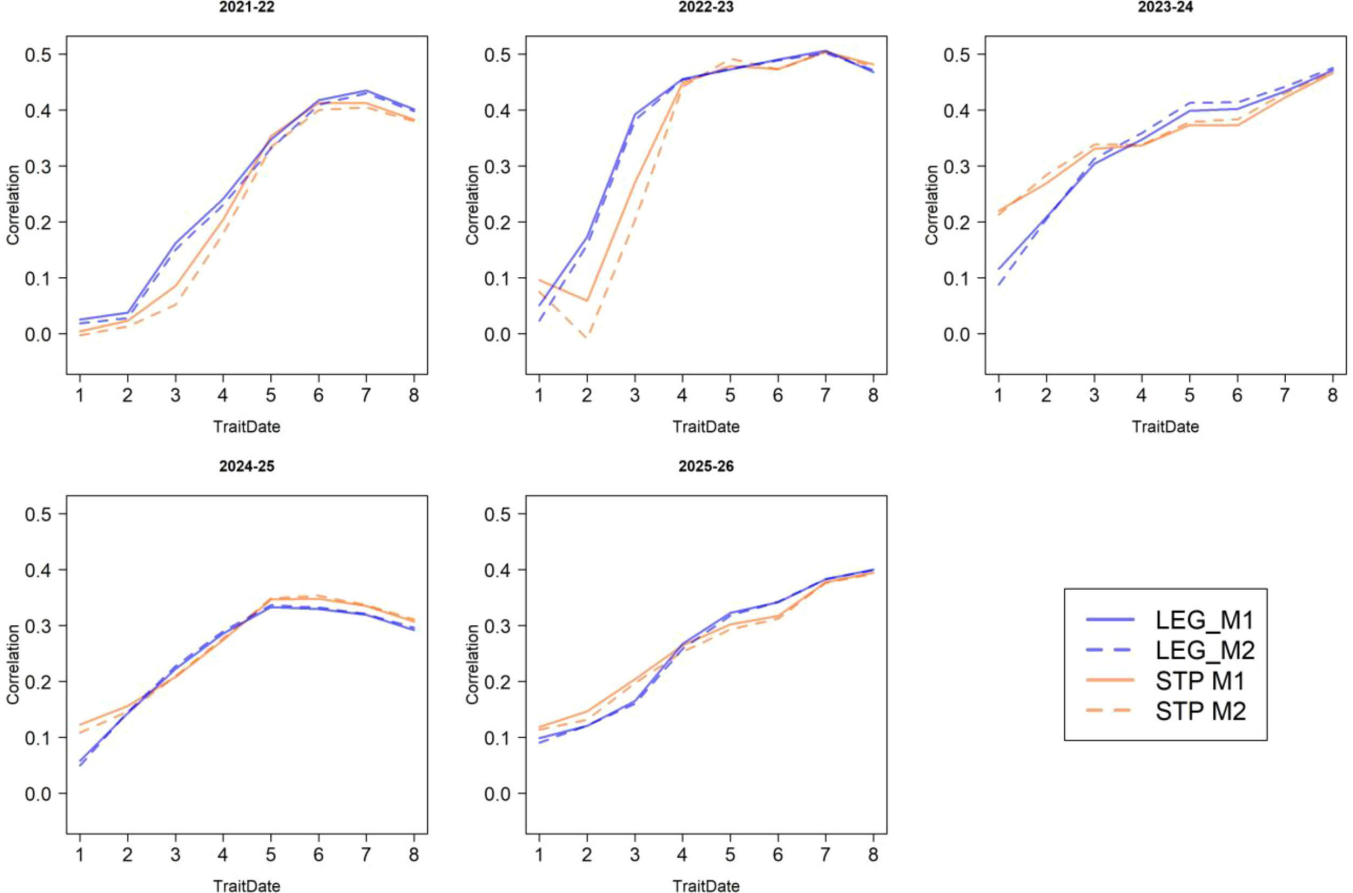
Predictive ability of Legendre and single time point models across fruiting weeks (trait dates 1–8) and five seasons for models M1 (Environment, Genotype and Marker main effects E+L+G) and M2 (also including the genotype-by-environment interaction effects E+L+G+ GE) for cumulative Marketable Yield. In legend, LEG represents the legendre approach and STP represents the single time point approach, where each trait date is modeled independently.

Across the five validation seasons, genomic selection predictive ability obtained from Legendre polynomials generally increased across the season, with the lowest values observed at Trait Date 1 and highest values occurring at later Trait Dates (**Figure 5**). When predicting the 2023–24 and 2025–26 season, predictive ability increased steadily across Trait Dates, reaching its highest value at Trait Date 8 for both M1 and M2. On the other hand, when predicting the 2021-22, 2022–23 and 2024–25 season, predictive ability increased across Trait Dates, reaching its highest values at Trait Date 6, followed by a slight decline at Trait Date 8. In 2021–22 and 2022-23, predictive ability rose sharply between Trait Dates 2 and 4–5 and then plateaued or declined slightly at the final Trait Dates.

In 2021–22 and 2022–23 seasons, Legendre models showed higher predictive ability than single time point models at most Trait Dates, with steeper gains from early harvests and higher peaks before the final Trait Dates. In 2023-24, 2024-25, and 2025-26, Legendre and single time point trajectories were closely aligned, and differences in predictive ability were small at nearly all Trait Dates. In 2023–24 and 2025-26, Legendre models showed slightly higher predictive ability from Trait Dates 4-8, but marginally lower values at the earliest Trait Dates. In 2024-25, predictive ability was more closely aligned between the two approaches, with Legendre showing marginally higher values at Trait Dates 3 and 4 and slightly lower values at Trait Date 1. In 2024–25 and 2025-26, maximum predictive ability remained lower than in other seasons, regardless of the model and approach, whereas in 2021-22, 2022-23, and 2023–24 higher plateaus were reached by later Trait Dates.

Within each modeling framework, the two genomic prediction specifications, M1 and M2 showed very similar predictive ability. For both Legendre and single time point models, M1 and M2 trajectories largely overlapped across Trait Dates in all five seasons. Only minor deviations were observed at isolated time points, and no consistent advantage of one specification over the other was evident.

For the Legendre predicted coefficients, the lower-order terms ( $\beta_{0}$ and $\beta_{1}$) consistently showed the highest predictive ability across all seasons (**Table 2**). The second-order coefficient ( $\beta_{2}$) exhibited moderate predictive ability, while the third-order coefficient ( $\beta_{3}$) had the lowest values. This ranking was stable across both the models and across all validation seasons, with $\beta_{0}$ and $\beta_{1}$ outperforming the higher-order coefficients in every season.

**Table 2:** Predictive ability of Legendre polynomial coefficients ( $\beta_{0,}\;\beta_{1,}\;\beta_{2,}\;$and $\;\beta_{3}$) for cumulative Marketable Yield under two genomic prediction models (M1 = E+L+G; M2 = E+L+G +GE) across five seasons.

Season	Model	$\beta_{0}$	$\beta_{1}$	$\beta_{2}$	$\beta_{3}$
2021-22	E+L+G	0.42	0.43	0.26	0.13
	E+L+G+GE	0.40	0.43	0.26	0.12
2022-23	E+L+G	0.52	0.49	0.28	-0.01
	E+L+G+GE	0.51	0.49	0.28	-0.03
2023-24	E+L+G	0.44	0.41	0.27	0.12
	E+L+G+GE	0.45	0.42	0.28	0.14
2024-25	E+L+G	0.33	0.29	0.15	0.04
	E+L+G+GE	0.33	0.29	0.15	0.04
2025-26	E+L+G	0.37	0.32	0.04	0.05
	E+L+G+GE	0.36	0.33	0.05	0.13

Values represent the correlation between observed and predicted coefficients in the 2021-22, 2022-23, 2023-24, 2024-25, and 2025–26 seasons.

**Figure 6:**
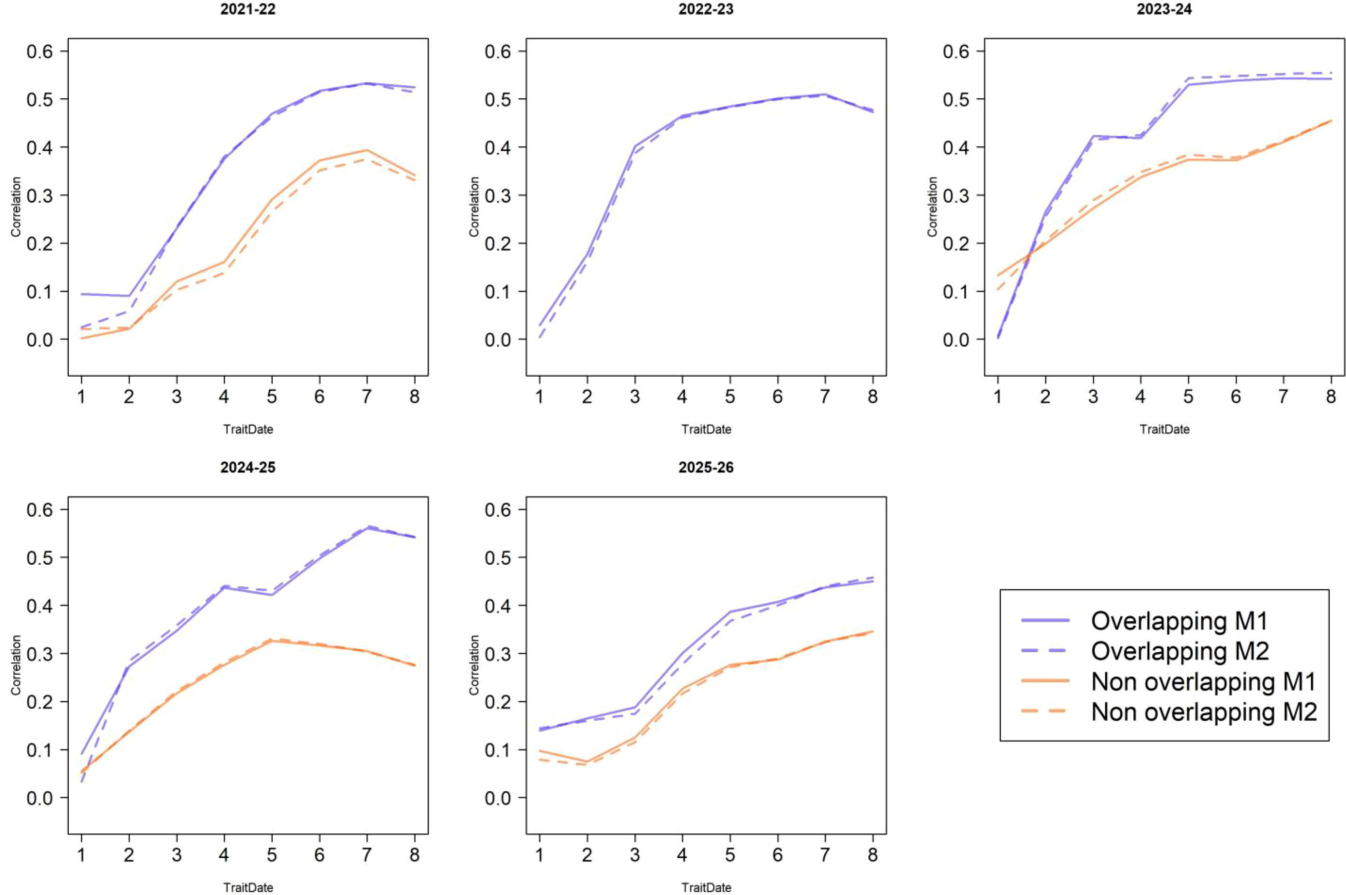
Predictive ability for overlapping (genotypes present in both training and validation sets) and non-overlapping (previously unseen) genotypes across Trait Dates in the 2021–22, 2022–23, 2023–24, 2024–25, and 2025–26 seasons based on Legendre polynomial predictions.

Predictive performance in forward prediction is influenced by the degree of overlap between training and validation genotypes. For the Legendre-predicted values, correlations were evaluated separately for genotypes that were present in both the training and validation sets (overlapping) and for genotypes that were newly evaluated in the validation season (non-overlapping). The number of overlapping and non-overlapping genotypes differed across seasons. In 2025-26, there were 257 overlapping genotypes and 756 non-overlapping genotypes. In 2024-25, 58 genotypes overlapped with previous seasons, whereas 860 were new. In 2023–24 there were 67 overlapping and 318 non-overlapping genotypes. In 2022–23 most genotypes overlapped with previous seasons (435), and only 12 non-overlapping genotypes. In 2021–22 there were 106 overlapping and 345 non-overlapping genotypes. Across all seasons, predictive ability was consistently higher for overlapping genotypes than for non-overlapping genotypes (**Figure 6**). In 2021-22, predictive ability increased steadily through the early Trait Dates, with the highest values observed around Trait Date 8 for both groups: approximately 0.52 for overlapping genotypes and 0.39 for non-overlapping genotypes. The trajectories of non-overlapping genotype predictions are not shown for the 2022–23 season because only a small number of genotypes (n = 12) were unique to that season. This limited sample size was insufficient to support a meaningful assessment of predictive ability and was therefore excluded to avoid over-interpretation. Predictive ability for overlapping genotypes remained positive across all Trait Dates and increased steadily, reaching its highest value (r = 0.51) at Trait Date 7. In 2023-24, predictive ability for the two groups was similar at Trait Date 2, but clear separation emerged as the season progressed. In 2024-25, predictive ability increased progressively across Trait Dates for both genotype groups with maximum correlation observed at Trait Date 7 for overlapping genotypes while at Trait Date 5 for non-overlapping. In 2025-26, a similar trend was observed. The highest predictive ability for overlapping genotypes reached approximately 0.46 at Trait Date 8, whereas non-overlapping genotypes achieved lower values, with maximum of 0.35.

**Figure 7:**
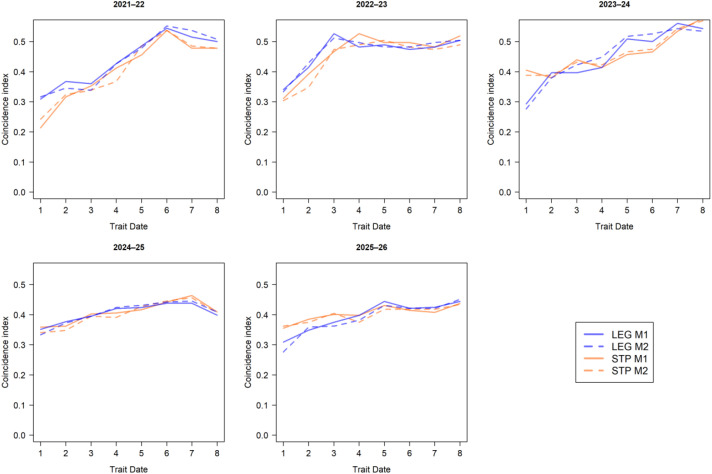
Coincidence index (top 30%) comparing model performance based on cumulative marketable yield across 8 trait dates and five validation seasons for models M1 (Environment, Genotype and Marker main effects E+L+G) and M2 (also including the genotype-by-environment interaction effects E+L+G+GE). In legend, LEG represents the legendre approach and STP represents the single time point approach, where each trait date is modeled independently.

Across the five validation seasons, both Legendre polynomial and single time point models showed similar overall trends in coincidence index, with values generally increasing as the season progressed (**Figure 7**). In 2022-23, differences between the two approaches were small, with Legendre outperforming at early Trait Dates and showing similar performance to single time point models after Trait Date 4. In 2023-24, Legendre models outperformed STP models, primarily between Trait Dates 4 and 7. In 2024–25 and 2025-26, Legendre and single time point models tracked very closely throughout the season.

#### (ii) Delta yield

**Figure 8:**
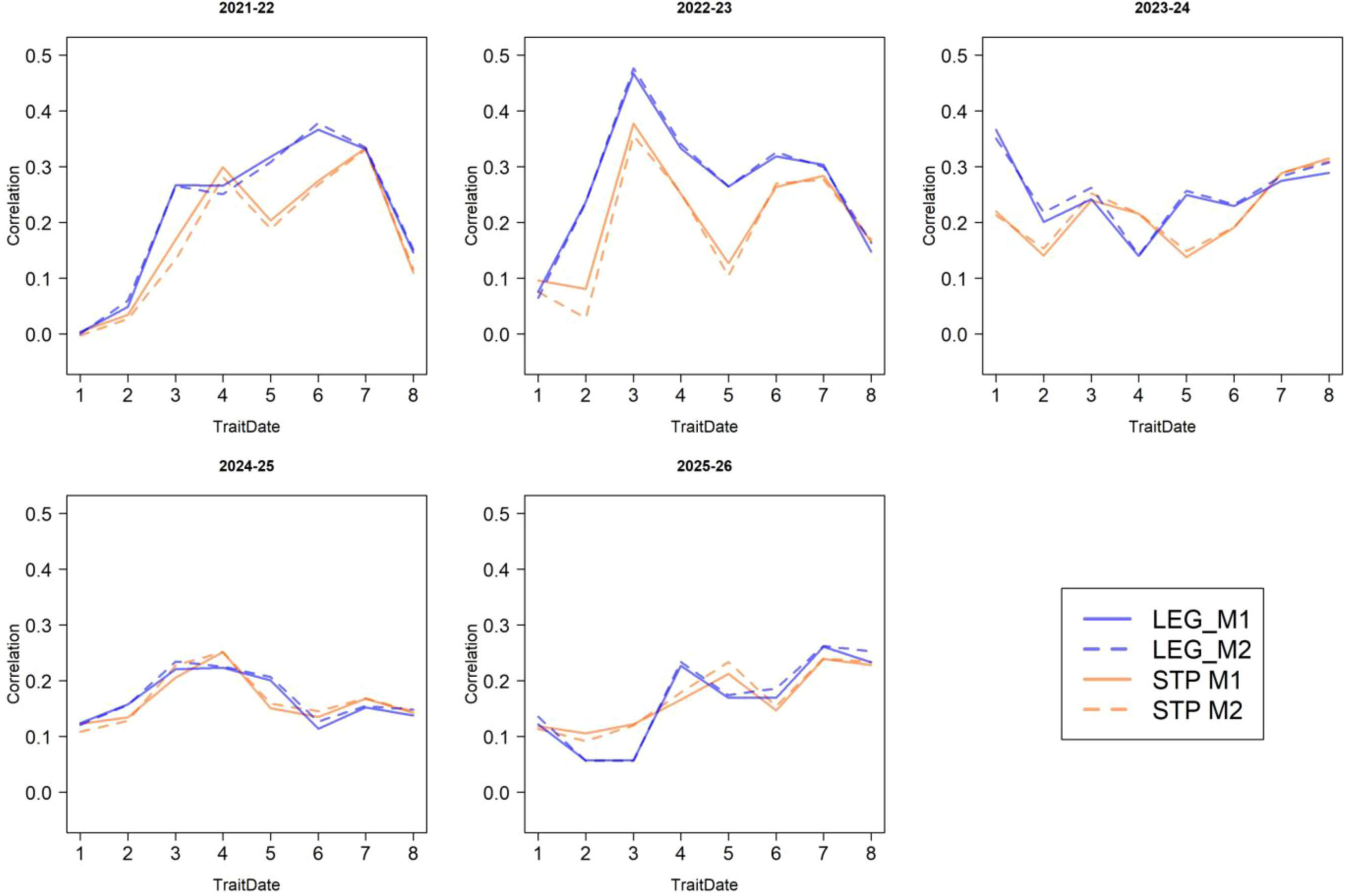
Predictive ability of Legendre and single time point models across fruiting weeks (Trait Dates 1-8) and five seasons for models M1 (Environment, Genotype and Marker main effects E+L+G) and M2 (also including the genotype-by-environment interaction effects E+L+G+ GE) for Delta Yield. In legend, LEG represents the legendre approach and STP represents the single time point approach, where each trait date is modeled independently.

Across the five validation seasons, genomic selection predictive ability obtained from Legendre polynomials showed variable trajectories depending on the season (**Figure 8**). In 2021-22, predictive ability rose steadily from Trait Date 1, peaking around Trait Dates 6–7 before declining slightly at Trait Date 8. In 2022-23, predictive ability increased sharply between Trait Dates 2 and 3, reaching the highest peak observed across all seasons, followed by a decline and a secondary plateau from Trait Dates 5–7 before dropping again at Trait Date 8. In 2023-24, predictive ability began relatively high at Trait Date 1, fluctuated modestly across intermediate Trait Dates, and converged to moderate values by Trait Date 8. In 2024–25 and 2025-26, predictive ability followed flatter trajectories overall, with a modest rise peaking around Trait Dates 3-4 and then stabilizing at lower values through Trait Date 8.

Across most seasons, Legendre polynomial models and single time point models tracked closely, though meaningful differences emerged in specific seasons. In 2022-23, Legendre models showed notably higher predictive ability than single time point models at early Trait Dates, particularly at Trait Dates 1-3, where the gap was most pronounced before the two approaches narrowed by Trait Dates 6-8. In 2021-22, the Legendre models showed marginally higher values at Trait Dates 2-7. In 2023-24, 2024-25, and 2025-26, Legendre and single time point trajectories were nearly indistinguishable at most Trait Dates, with only minor differences at select intermediate points. Notably, maximum predictive ability was substantially higher in 2022–23 and 2021–22 relative to the remaining seasons, whereas 2024–25 and 2025–26 showed the lowest overall predictive ability regardless of model or approach.

**Figure 9:**
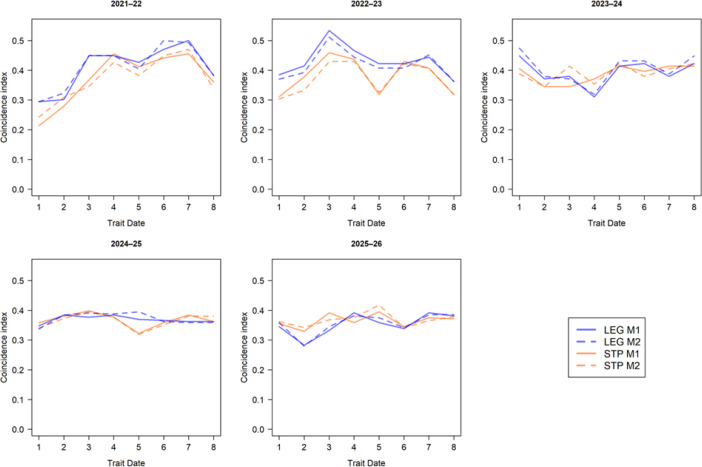
Coincidence index (top 30%) comparing model performance based on delta yield across 8 trait dates and five validation seasons for models M1 (Environment, Genotype, and Marker main effects E+L+G) and M2 (also including the genotype-by-environment interaction effects E+L+G +GE).

Across the five validation seasons, Legendre polynomial models generally showed higher or equal coincidence index values than single time point models, though differences varied by season (**Figure 9**). In 2021–22 and 2022-23, Legendre models consistently outperformed single time point models. In 2023-24, differences between the two approaches were small across most Trait Dates, with Legendre showing only marginally higher values at earlier Trait Dates before narrowing toward Trait Date 8. In 2024–25 and 2025-26, Legendre and single time point models tracked very closely throughout the season.

## Discussion

### Leveraging longitudinal data for breeding decisions

Breeding programs often have access to historical datasets that can be used for predicting future phenotypes. In crops with repeated harvests, such as strawberries, yield is recorded across multiple picking dates rather than at a single terminal harvest. This longitudinal structure offers unique insights into how genetic effects unfold over time. This temporal expression is highly relevant for Florida strawberry production, where early season yield in December and January is highly profitable due to premium market prices early season (Whitaker et al., 2017; Peres et al., 2006). Economic analyses show that increasing yield during the first 6–8 weeks of the harvest season provides the greatest benefit to Florida strawberry growers (Wu et al., 2015). Thus, targeting early yield is likely to generate more economic returns, even if total seasonal yield remains unchanged.

To address this, we analyzed past multi-year strawberry yield datasets using Legendre polynomial regression to model yield trajectories to predict the performance in the most recent seasons. Legendre polynomials were chosen over logistic or any other biological functions because of their flexibility to model yield without assuming fixed sigmoidal shapes which do not always follow a smooth pattern (Archontoulis and Miguez, 2015). On the other hand, Legendre polynomials do not assume a specific curve shape. Modeling yield as a continuous function allows the framework to interpolate between picking events and normalize responses when harvests are uneven. This is especially useful in scenarios where data collection is irregular, such as when weather disrupts picking schedules, when historical datasets vary in harvest frequency, or in crops like forages where harvests are inherently sporadic.

### Stability of the Legendre polynomial framework

The Legendre polynomial framework models yield as a continuous trajectory over time, allowing information to be shared across neighboring trait dates (Momen et al., 2019). By modeling yield as a continuous trajectory rather than a series of independent observations, the approach provides a more coherent representation of genotype performance over time. Such continuity mitigates the random noise inherent to individual harvest estimates and better delineates genotype-specific growth dynamics. From a breeding perspective, this framework is particularly useful for predicting the full yield curves of genotypes using historical genomic and phenotypic information. Such predictions can help breeders anticipate the seasonal yield profiles of new or untested genotypes and prioritize those with desirable production patterns for field evaluation in subsequent seasons. Compared with single time-point estimates, the Legendre approach for cumulative marketable yield delivered higher predictive ability for 2021–22 and 2022-23. For 2023-24, Legendre approach performed better at later Trait Dates. For the 2024–25 and 2025-26, performance was similar for both approaches, indicating season specific variability that may be influenced by limited genotype overlap and differences in replication across years, which can affect model stability and comparability across seasons. Although the Legendre framework is designed to leverage temporal structure, its advantage may be less pronounced when growth patterns are consistent and variability across environments is limited. Under such conditions, both approaches capture similar trends in genotype performance. In contrast, greater benefits from functional approaches may be expected in more heterogeneous systems, where temporal dynamics differ more substantially across environments or genotypes. This likely explains the similar performance observed between Legendre and STP models in some validation seasons in this study, despite the theoretical advantages of longitudinal modeling.

From a methodological perspective, functional regression consistently provides advantages by leveraging full yield trajectories and minimizing bias. For breeders, this smoother trajectory reduces the risk of misleading conclusions from transient noise or measurement error. These results align with earlier studies showing that longitudinal data approaches improve the robustness of genomic prediction by capturing the continuity of developmental processes (Momen et al., 2019; de Souza et al., 2020; Campbell et al., 2018; Matsui and Mochida, 2024), although gains in predictive ability may depend on the structure and variability of the dataset.

### Interpreting Legendre coefficients

Unlike nonlinear biological functions such as logistic or Gompertz, where parameters (e.g., asymptote, growth rate, inflection point) carry direct biological meaning, Legendre polynomial coefficients are not individually interpretable in a straightforward biological sense. Instead, they are best understood collectively, as each coefficient contributes to shaping different aspects of the yield trajectory. For example, $\beta_{0}$ represents the baseline level of the curve, $\beta_{1}$ primarily influences the overall slope, while higher-order coefficients ( $\beta_{2}$ and $\;\beta_{3})\;$ capture curvature and sigmoidal features of the trajectory. This can be seen in **Supplementary Figure 2**, where increasing or decreasing each coefficient keeping others constant shifts the yield curve in distinct ways. However, from a breeder’s perspective, interpreting coefficients directly without reconstructing the entire curve can still provide useful insights. For instance, higher values of $\beta_{0}$​ and $\beta_{1}\;$combined with lower $\beta_{2}$ and $\beta_{3}\;$ generally correspond to genotypes with stronger early accumulation and sustained yield potential. This is illustrated in **Supplementary Figure 3**, where genotypic differences in Legendre coefficient patterns (radar plot) translate into distinct yield trajectories (functional curves).

### Model performance

Across the five forward prediction cases, the performance of the two different models (E+L+G and E+L+G+GE) was very similar. Examination of variance component estimates further indicated that the contribution of the G×E to the total variance in M2 was relatively very small. Several factors may explain this outcome. First, all trials were conducted within a single geographic region, so the environmental variation was limited to different seasons. The environmental effect in this study primarily represents year-to-year variability rather than spatial environmental heterogeneity. Such restricted environmental heterogeneity often reduces the opportunity to detect strong G×E effects (Rebollo et al., 2023; Monteverde et al., 2019). Werner (2022) also demonstrated that field trials limited to a single region constrain the environmental gradient, which often reduces power to characterize G×E interactions and results in environments primarily represented by year differences. Second, the dataset was unbalanced across years due to the routine removal of non-advanced lines and introduction of new genotypes. This reduced line connectivity across years can bias variance component estimates downward, as fewer observed genotype × year combinations limit the model’s ability to capture interaction effects (Laidig et al., 2008; Aguate et al., 2019). Future studies should evaluate these longitudinal genomic prediction models using multi-location and more environmentally diverse datasets to better determine the contribution of G×E interaction and assess whether incorporating G×E terms improves prediction performance across broader environmental conditions.

Across all five forward-prediction scenarios, predictive ability for cumulative marketable yield followed a consistent pattern: it started low at the earliest trait dates and gradually increased, reaching approximately 40-50% percent once more temporal information accumulated. Predicting genotypes that the model has already seen improved predictive ability, and the additional training data from the first season strengthened the model’s ability to capture stable genetic patterns. As the dataset expanded across years, the increasing volume of phenotypic and genotypic information strengthened the model’s underlying structure, and the predictions for 2023–24 aligned closely with the pattern observed in the preceding seasons.

Predictive performance in forward prediction is influenced by the degree of overlap between training and validation genotypes. Results showed higher correlation for the genotypes that were observed earlier (overlapping) compared to the newly introduced genotypes (non-overlapping). When a genotype has been evaluated in earlier seasons, its direct phenotypic records help define its expected performance in subsequent years. Newly introduced genotypes lack this historical information, so their predictions rely only on genomic relationships. Consequently, predictive ability is reduced for newly introduced genotypes under true forward-prediction scenarios. In the 2022–23 season, predictive ability estimates were sensitive to sample size. When correlations are computed on only a few genotypes with a narrow phenotypic range, the estimates become unstable and can fluctuate strongly (Lynch and Walsh, 1998).

### Temporal patterns in prediction ability

Reconstruction of the yield trajectories revealed clear temporal patterns in prediction ability. Both models exhibited low predictive ability during the early stages of the season but improved later with performance stabilizing around week 4. This pattern reflects the limited amount of yield accumulated during early harvests, where only a small fraction of total seasonal yield is expressed. As a result, the genetic signal is weak relative to noise, leading to lower prediction ability in early weeks. In contrast, later harvests (Trait Dates 4–8) capture a substantially larger proportion of total yield, integrating cumulative growth and canopy development, which are more strongly associated with genetic differences among genotypes (Ziliani et al., 2022; Tagarakis and Ketterings, 2017).

The instability observed during early trait dates is therefore likely driven by a combination of low yield magnitude and higher relative environmental and stochastic variation when fruit numbers are still limited. Under these conditions, small measurement errors or environmental fluctuations can disproportionately influence observed values, reducing the signal-to-noise ratio (Rutkoski et al., 2016; Habibi et al., 2024). Similar temporal pattern have been observed for phenomic selection based on UAV derived canopy traits (Sleper et al., 2025). From a breeding perspective, achieving reliable predictions by week 4 is highly valuable, as it could enable selections or crossing decisions several weeks earlier than waiting until week 8.

### Delta yield as a phenotypic trait for genomic selection

To our knowledge, this study is the first to apply Delta Yield as a phenotypic trait for genomic selection in strawberry breeding. By decomposing total marketable yield into discrete harvest intervals, this approach captures the genuine genetic signal expressed at each specific point in time rather than accumulated yield increasingly influenced by prior harvests. A fundamental limitation of cumulative yield in random regression modeling is that its inherently smooth trajectory is largely an artifact of progressive accumulation, obscuring true temporal variation in genetic expression. Delta Yield constrains information to each individual harvest interval, making it a more appropriate input for Legendre polynomial models designed to borrow information across time points and model the underlying genetic covariance structure of a trajectory.

Delta Yield-based genomic prediction achieved moderate to high predictive ability across all five validation seasons, with the strongest signals observed at intermediate harvest dates when fruiting dynamics were most actively expressed. By evaluating each harvest interval relative to the previous one, Delta Yield reduces the confounding influence of early-season establishment variation, more precisely isolating genetic differences in fruiting rate and inter-harvest recovery. This capacity to resolve genotypic performance within specific seasonal windows is particularly relevant for identifying cultivars suited to early or late season production, carrying considerable importance for regional production systems and market timing (Whitaker et al., 2012). From a grower perspective, varieties that deliver steady, distributed yield across the season are preferable to those producing a single concentrated flush of fruit, as consistent harvest intervals improve labor scheduling, reduce post-harvest losses, and stabilize market supply during premium-price windows. Beyond strawberry, the capacity to model and select for yield trajectory consistency may prove broadly applicable to other indeterminate horticultural crops, including tomato, pepper, and blueberry, where repeated fruiting cycles, perishability, and market timing similarly constrain profitability and where genomic tools for capturing temporal yield dynamics remain largely underdeveloped. However, because Delta Yield is inherently sensitive to short-term environmental and operational variability, further validation across broader environments and breeding populations is needed before it can be reliably implemented as a routine genomic selection trait.

### Broader implications and future directions

This study highlights the value of integrating trajectory-based genomic prediction approaches, such as Legendre polynomial regression, into breeding pipelines for crops with longitudinal yield data. In crops like strawberries, breeding decisions operate within narrow seasonal windows for crossing, seed extraction, germination, and seedling establishment. Delays in decision making can directly translate into lost opportunities for advancing superior genotypes. In this context, the ability to obtain reliable predictions several weeks earlier (e.g., around Trait Date 4 rather than at the end of the season) can substantially improve breeding efficiency. Earlier information allows breeders to make timely crossing and selection decisions within the same season, effectively shortening the breeding cycle and increasing the rate of genetic gain.

Although early season predictions may not be fully optimal, they are still informative and can improve decision making relative to relying solely on end-of-season data. This aligns with a Bayesian perspective in breeding, where decisions are continuously updated as new data becomes available. Incorporating in-season predictions enables breeders to refine selection decisions using the most current information, thereby improving the overall efficiency of the breeding program.

While both trajectory modeling and crop growth models aim to capture yield as a dynamic process, they differ in approach: Legendre regression is statistical and flexible, requiring no prior assumptions, whereas crop growth models are mechanistic and rely on biological inputs. The former remains data-driven and unbiased, while the latter offers biological interpretability but can be biased if assumptions are inaccurate. Future work could explore integrating these frameworks to combine predictive performance with biological insight.

Additional research is also needed to evaluate whether reduced sampling frequency (e.g., biweekly harvests) can maintain predictive accuracy, and whether early or short-term forecasts can reliably predict final yield. Moreover, incorporating environmental covariates (e.g., temperature, radiation, and humidity) directly into the longitudinal models would enable forecasting of yield trajectories, both within and across locations, and facilitate scenario analyses under alternative weather patterns. Such extensions would also support prediction of on-farm yield, providing growers with operationally relevant estimates of expected production that could inform decisions on storage capacity, labor allocation, and distribution logistics.

Beyond strawberries, the methodology can be applied to other crops with repeated harvests (e.g., tomatoes, peppers, etc.) or longitudinal phenotyping (e.g., forage grasses), offering breeders a more powerful tool to exploit multi-year datasets and accelerate genetic gains. This work also contributes to bridging the gap between advanced statistical modeling and practical breeding decisions, supporting the development of cultivars that are both high yielding and better adapted to changing environmental conditions.

## Conclusions

This research advances genomic prediction by demonstrating the utility of longitudinal modeling approaches for capturing trait dynamics over time. Using Legendre polynomial regression, yield trajectories were modeled as continuous functions, reducing noise associated with discrete, week-by-week predictions and providing a more reliable picture of temporal genetic effects. The methodological contribution lies in showing that longitudinal models capture the continuity of crop development more effectively than single time point-based models, offering breeders tools to identify when and how genetic signals emerge during the season. The practical significance is particularly evident in strawberries, where early season yield strongly influences profitability. Also, reliable predictions by Week 4–5 instead of Week 8 give breeders an earlier indication of genotype performance, and an extra month to select crosses or make advancement decisions, with direct implications for breeding efficiency. By linking genetic coefficients to yield dynamics, this approach supports earlier, more informed selection decisions and provides a foundation for integrating economic considerations into breeding pipelines. These findings illustrate the broader potential of longitudinal genomic prediction across crops and environments. As breeding programs increasingly generate multi-year, high-throughput, and temporally rich datasets, models that can handle temporal elements will be essential.

## Data Availability

The datasets presented in this study can be found in online repositories. The names of the repository/repositories and accession number(s) can be found in the article/**Supplementary Material**.
