# Supplementary material for: Modeling production curves in a strawberry breeding program to optimize early season productivity
Source: Front Plant Sci. 2026 Jul 16;17:1808529. doi: 10.3389/fpls.2026.1808529 (PMC13420405; doi:10.3389/fpls.2026.1808529)
Supplement: Supplementary file 1 [file DataSheet1.docx]

Supplementary figures and tables


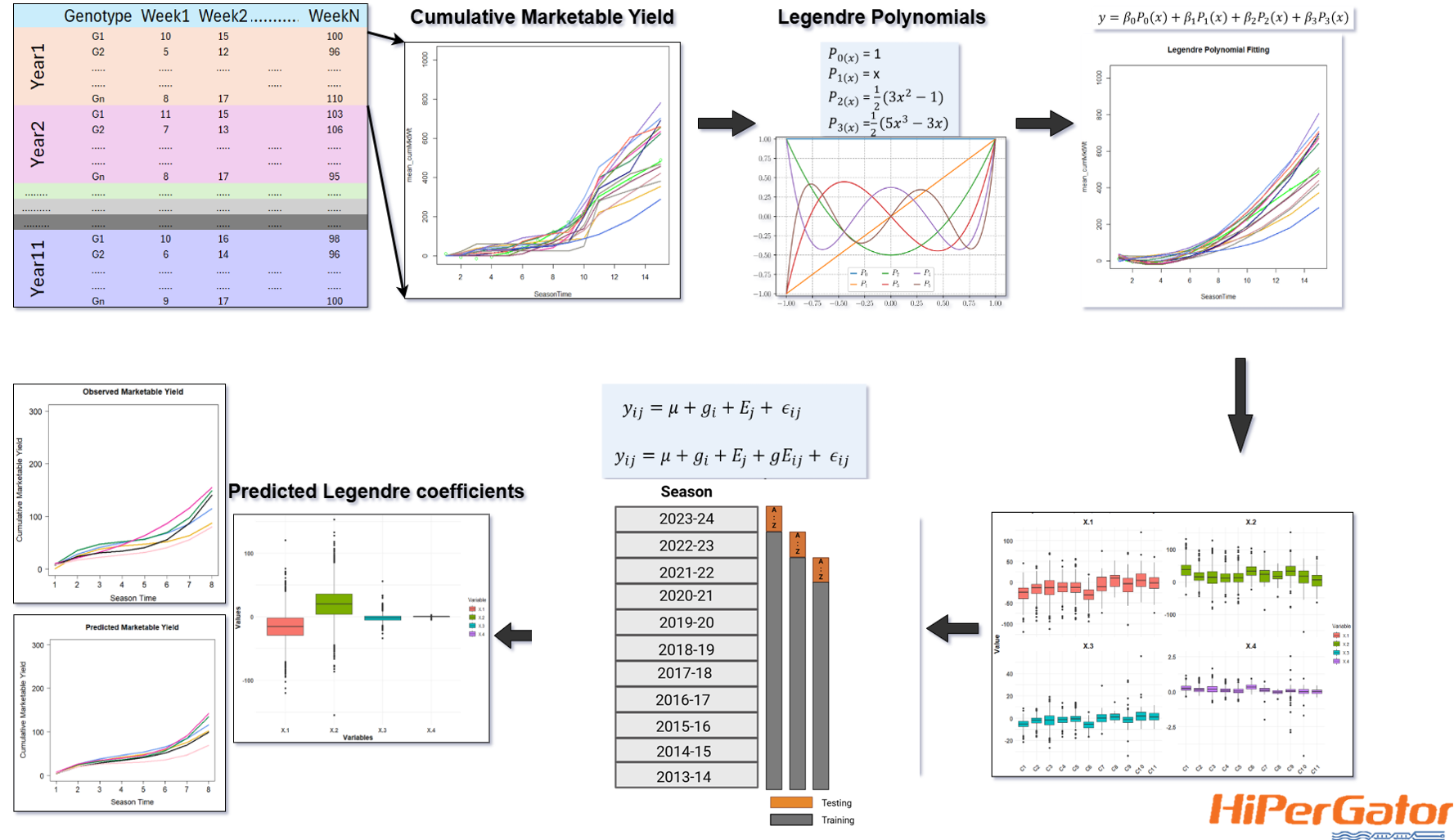


**Supplementary Fig. 1:** Workflow for modeling cumulative marketable yield trajectories using Legendre polynomials in multi-year trials: Weekly yield data across 11 years were expressed as growth trajectories and fitted with Legendre polynomials $(\beta_{o},\beta_{1},\beta_{2}, \beta_{3}).$ The fitted coefficients were analyzed in a mixed model framework (M1 and M2) with genotype, environment, and G×E effects. Two cross-validation schemes (within known and unknown environments) were considered, and predicted coefficients are used to reconstruct yield curves.


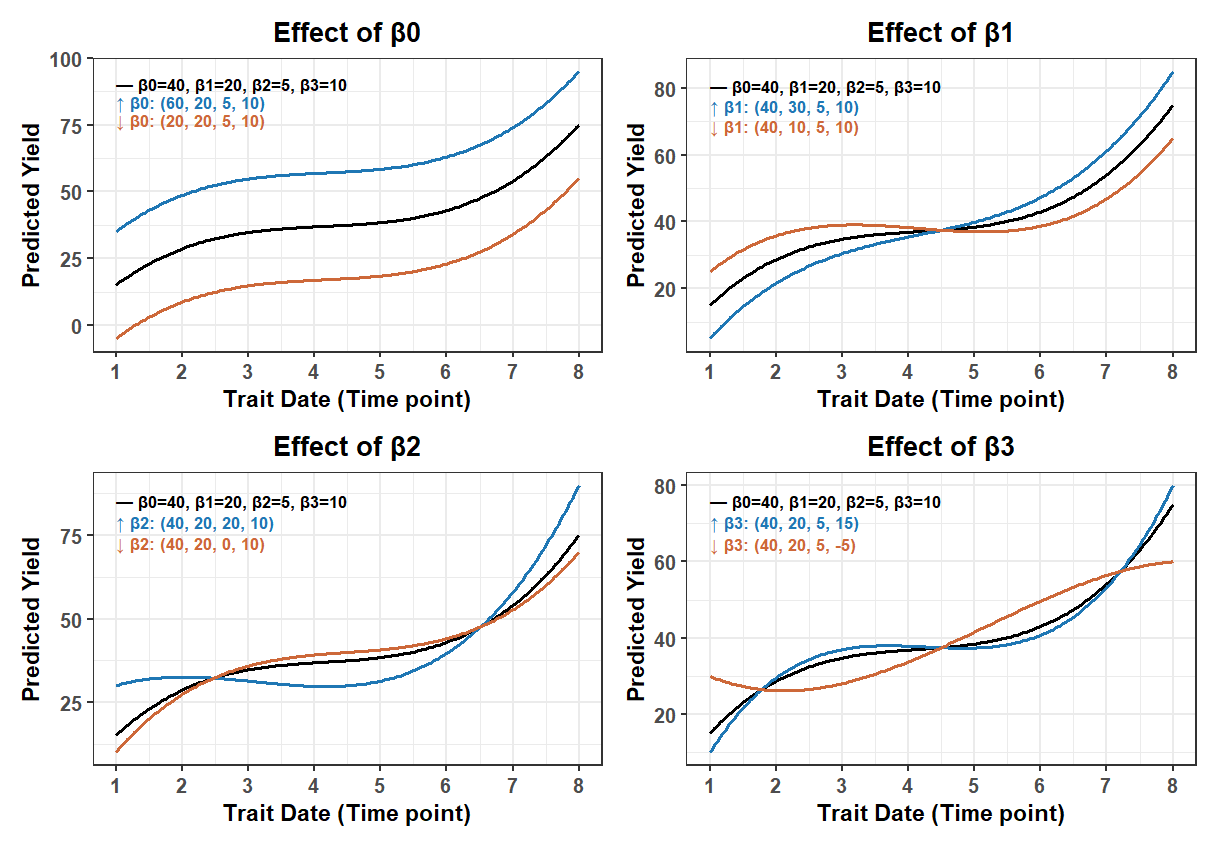


**Supplementary** **Fig. 2:** Effect of Legendre Polynomial Coefficients (${\beta_{0}, \beta}_{1}, \beta_{2}, {and \beta}_{3}$) on Predicted Yield Trajectories Across Scaled Season Time.


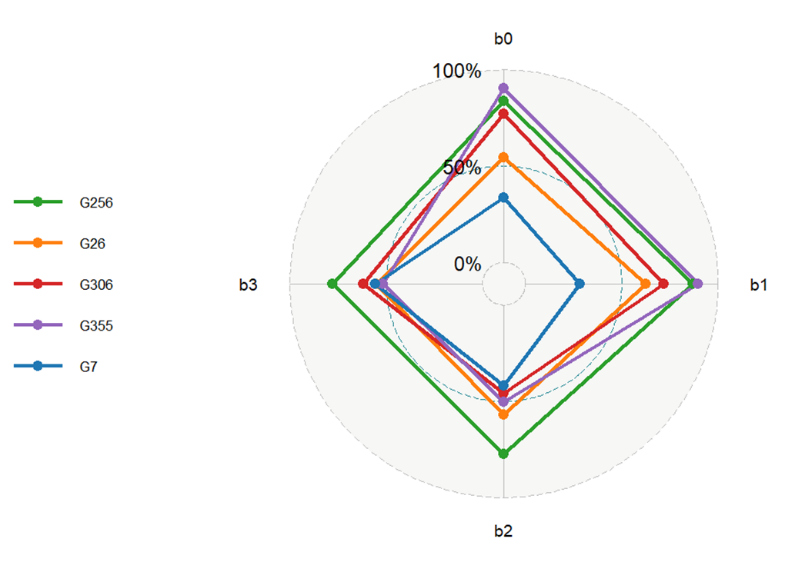

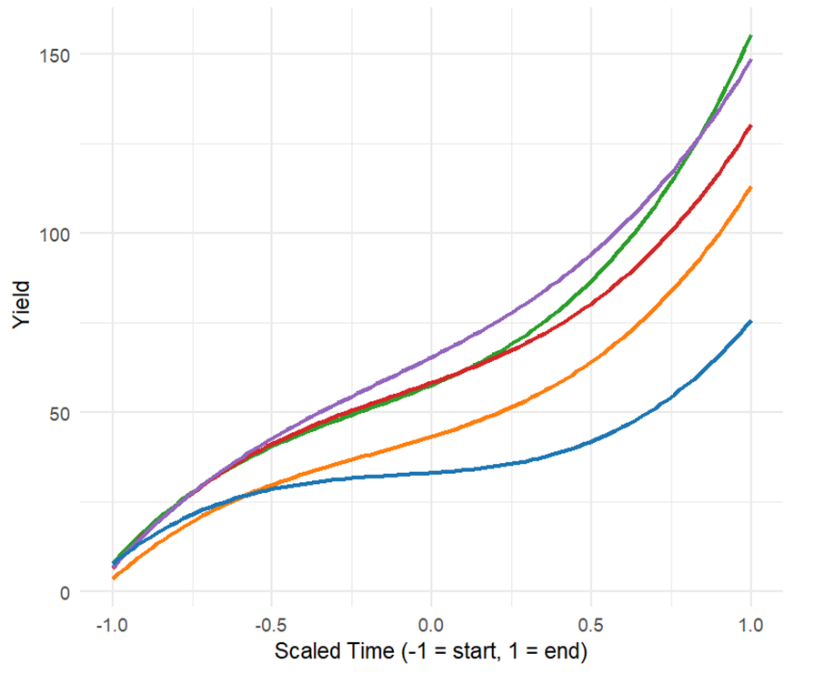


**Supplementary** **Fig. 3** Genotype performance across time was summarized using Legendre coefficient profiles and corresponding predicted yield trajectories, visualized through radar plots and functional curves (Supplementary Fig. 3).

**Supplementary Table** **1:** Summary of trait dates, calendar timing, season time, and classification into extra early (EE), mid early (ME), and early (E) stages for the 2023-24 season.

| **Trait Date** | **Calendar Date** | **Season Time (DAP)** | **Stage** |
| --- | --- | --- | --- |
| 1 | 28 Nov | 49 | EE |
| 2 | 5 Dec | 56 | EE |
| 3 | 12 Dec | 63 | EE |
| 4 | 19 Dec | 70 | ME |
| 5 | 26 Dec | 77 | ME |
| 6 | 2 Jan | 84 | ME |
| 7 | 9 Jan | 91 | E |
| 8 | 16 Jan | 98 | E |
